# Prevalence and determinants of subretinal drusenoid deposits in patients’ first-degree relatives

**DOI:** 10.1007/s00417-023-06221-y

**Published:** 2023-09-06

**Authors:** Matthias M. Mauschitz, Benedikt J. Hochbein, Hannah Klinkhammer, Marlene Saßmannshausen, Jan H. Terheyden, Peter Krawitz, Robert P. Finger

**Affiliations:** 1 Department of Ophthalmology, University Bonn, Ernst-Abbe-Straße 2, 53127 Bonn, Germany; 2 Institute for Genomic Statistics and Bioinformatics, University Bonn, Bonn, Germany; 3 Institute for Medical Biometry, Informatics and Epidemiology, University Bonn, Bonn, Germany

**Keywords:** Subretinal drusenoid deposits, Genetics, Age-related macular degeneration, Imaging

## Abstract

**Purpose:**

Subretinal drusenoid deposits (SDDs) are distinct extracellular alteration anterior to the retinal pigment epithelium (RPE). Given their commonly uniform phenotype, a hereditary predisposition seems likely. Hence, we aim to investigate prevalence and determinants in patients’ first-degree relatives.

**Methods:**

We recruited SDD outpatients at their visits to our clinic and invited their relatives. We performed a full ophthalmic examination including spectral domain–optical coherence tomography (SD-OCT) and graded presence, disease stage of SDD as well as percentage of infrared (IR) en face area affected by SDD. Moreover, we performed genetic sequencing and calculated a polygenic risk score (PRS) for AMD. We conducted multivariable regression models to assess potential determinants of SDD and associations of SDD with PRS.

**Results:**

We included 195 participants, 123 patients (mean age 81.4 ± 7.2 years) and 72 relatives (mean age 52.2 ± 14.2 years), of which 7 presented SDD, resulting in a prevalence of 9.7%. We found older age to be associated with SDD presence and area in the total cohort and a borderline association of higher body mass index (BMI) with SDD presence in the relatives. Individuals with SDD tended to have a higher PRS, which, however, was not statistically significant in the multivariable regression.

**Conclusion:**

Our study indicates a potential hereditary aspect of SDD and confirms the strong association with age. Based on our results, relatives of SDD patients ought to be closely monitored for retinal alterations, particularly at an older age. Further longitudinal studies with larger sample size and older relatives are needed to confirm or refute our findings.

**Supplementary Information:**

The online version contains supplementary material available at 10.1007/s00417-023-06221-y.


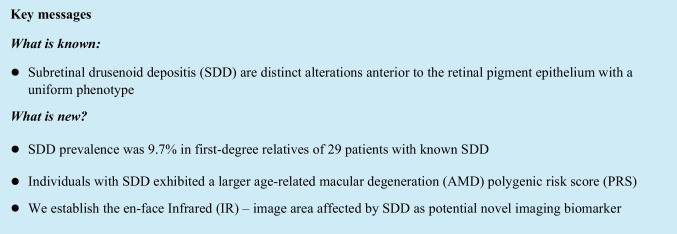


## Introduction

The outer retina is a metabolically highly active tissue with a large turnover of lipids in the visual cycle of the photoreceptors and resulting degradation products. Drusen represent focal accumulations of metabolic by-products including lipids and lipoproteins on the inner surface of Bruch’s membrane (BM) posterior to the retinal pigment epithelium (RPE) and are a hallmark age-related macular degeneration (AMD) lesion [1, 2]. In contrast, subretinal drusenoid deposits (SDDs) also known as reticular pseudodrusen are distinct extracellular alterations of the retina anterior to the RPE monolayer and can occur isolated or in relation to another retinal pathology such as AMD or rare genetic diseases such as pseudoxanthoma elasticum (PXE) [3–8].

SDDs have been linked to different functional impairments such as scotopic vision impairments, and AMD patients with SDD have been reported at higher risk for conversion to late stage atrophic AMD [3–5, 9]. Previous studies have reported a stronger impairment of scotopic visual function (e.g., rod intercept time, dark adaptation time) in patients with SDD compared to phenotypes presenting with predominantly drusen. All these findings point at different underlying disease pathways in presence of SDD prior leading towards outer retinal atrophy compared to eyes without SDD [10]. However, overall prevalence estimates vary significantly from 0.41 to 18.1% between studies depending on imaging modalities [11–15].

Previously, older age, higher body mass index (BMI), smoking, cardiovascular risk factors, and thinner choroid have been reported as determinants of SDD [4, 11, 16, 17]. Moreover, specific genetic variants in the age-related maculopathy susceptibility 2 (ARMS2), complement factor H (CFH), and hepatic triglyceride lipase (LIPC) genes have been identified as potential genetic risk factors [16, 17], while lipid-lowering drugs such as statins seem to have a protective effect [13]. While the commonly uniform phenotype suggests a common genetic component to SDD, we know very little about the heritability to date as barely any data exist on the frequency of SDD in relatives of patients with SDD. Hence, we aim to evaluate prevalence and determinants of SDD in first-degree relatives of patients with SDD and the association with AMD-linked genetic polymorphisms.

## Methods

### Subjects

We recruited index patients with presence of SDD (dot and/or ribbon type), potentially with a mixed phenotype with conventional drusen, from the outpatient clinic of the university eye hospital Bonn. Eligible patients were invited to approach potential first-degree relatives for inclusion. Included first-degree relatives were scheduled for a separate appointment. We acquired two informed consents (IC), one general and one for genetic testing, from all patients and relatives. The study adhered to the tenets of the Declaration of Helsinki and had local ethical committee approval.

### Examination protocol

We acquired demographic characteristics and medical history data including medication, smoking habits, and history of ophthalmic and cardiovascular diseases through a standardized questionnaire. Refraction and best-corrected visual acuity (BCVA) were assessed using the NIDEK (ARK-560A, NIDEK, Japan) auto-refractometer, and intraocular pressure (IOP) was measured using a non-contact tonometer (Tomey FT-1000). BCVA and low-luminance visual acuity (LLVA) were assessed using a standardized ETDRS chart as described elsewhere [18].

Subsequently, all index patients received mydriatic eye drops (0.5% tropicamide and 2.5% phenylephrine) for their appointment in the outpatient clinic, whereas most relatives opted out of pupil dilatation at their appointment.

All participants underwent near-infrared (IR) and spectral domain–optical coherence tomography (SD-OCT) imaging of the macula in both eyes, using the SPECTRALIS OCT (Heidelberg Engineering, Germany) with a scan pattern of 121 B-scans at 30° × 25° and 25 automated real-time (ART) frames in high-resolution (HR) mode. In index patients with poor fixation due to low sharp and central vision, fewer B-scans with less ART frames and lower resolution were performed. Subsequently, all participants underwent a complete ophthalmic examination of both eyes by an ophthalmologist, including slit-lamp examination and fundoscopy. Lastly, blood samples were taken (two 7.5 ml ethylenediaminetetraacetic acid (EDTA) tubes) for genetic testing.

### Image grading

Grading for SDD was performed on en face IR and SD-OCT B-scan imaging by two experienced graders (BJH, MMM) based on previously reported criteria (network of roundish irregularities, hyperreflective abnormalities/elevations anterior to the RPE, minimum five lesions) with senior arbitration in cases of doubt (RPF) [6, 19–22]. Moreover, in case of sufficient image quality, we measured the percentage of area with present SDD on the en face 30° IR image adjusted for individual image size (supplemental figure 1). In addition, we assessed the presence of retinal lesions associated with AMD such as drusen, pigmentary abnormalities, geographic atrophy (GA), and macular neovascularization (MNV) based on SD-OCT.

### Statistical analysis of clinical data

We performed descriptive statistics with mean and standard deviation (SD) for normally distributed and median and interquartile-range (IQR) for non-normally distributed data and calculated the prevalence of SDD within the group of relatives. Subsequently, we conducted multivariable logistic regression models with prevalent SDD as dependent variable and controlled for potential determinants. Models were controlled for age, sex, body mass index (BMI), smoking status (never, former, current), spherical equivalent (SE), previous cataract surgery, and prevalence of hypertension and diabetes. Co-variables were chosen a priori on the basis of literature and availability [3, 12, 13]. Moreover, we performed multivariable linear regression models with the percentage of affected area as outcome controlling for the same potential confounders. All analyses were performed with the statistical software RStudio (version 4.0.2, R Core Team (2021). R: A language and environment for statistical computing. R Foundation for Statistical Computing, Vienna, Austria. URL: https://www.R-project.org/).

### Genetic analyses

Samples of 189 individuals (index patients and relatives who consented to genetic testing) were genotyped on an Illumina GSA array Infinium iSelect 24×1 HTS Custom Beadship. Genotypes were then imputed using a local pipeline based on the bioinformatic tool Minimac4 and haplotypes from the 1000 Genomes Project [23]. Quality control steps were performed including the exclusion of single nucleotide polymorphisms (SNPs) that have either an imputation quality of R^2 < 0.3, a genotype rate of less than 90% or a minor allele frequency of less than 0.1% or that fail the Hardy-Weinberg equilibrium. Finally, individuals with more than 10% missing genotype data were dismissed. To derive a polygenic risk score (PRS), 69 SNPs associated with AMD were used. These include among others loci affecting pathways of inflammation as well as the lipid metabolism [24]. Overall, 54 out of 69 considered SNPs as well as 165 individuals passed the quality control. Based on those SNPs, the PRS was calculated via plink2 (http://www.cog-genomics.org/plink/2.0/, [25]) using the effect estimates derived by Han et al. [24]. Finally, the PRS was scaled via *z*-score within the cohort resulting in the final AMD PRS.

### Statistical analysis of polygenic risk data

To investigate associations of the PRS and the AMD and SDD status of individuals, a generalized linear mixed model was fitted; AMD and SDD status was defined as binary response variable (yes/no; questionable cases were used as absent SDD) and regressed on the AMD PRS while adjusting for sex and age and considering the family ID as a random variable. As there were too few participants with SDD and without AMD (*N* = 7), we could not adjust our genetic SDD models for AMD status due to lack of statistical robustness.

Two further linear mixed models were fitted using the percentage of SDD in both eyes, respectively, as linear outcome variable and AMD PRS as a predictor and adjusting for sex, age, and AMD status. Family ID was again considered as a random variable. Data were available for 90 individuals for the right eye and 96 individuals for the left eye.

Lastly, SDD status of the individuals was regressed separately on each of the 54 SNPs included in the PRS via a generalized linear mixed model while controlling for sex and age. One SNP, for which the model did not converge, was discarded. Associations were checked for nominal significance (*α* = 0.05) as well as Bonferroni-corrected significance (*α* = 0.0009).

## Results

We included 195 participants, 123 index patients (mean age 81.4 ± 7.2 years; 67% women) and 72 first-degree relatives (mean age 52.2 ± 14.2 years; 56% women). While one index patient had PXE, the majority (94%) had neovascular AMD, which was treated with intravitreal injections in at least one eye.

We found present SDD in 7 of 72 first-degree relatives with a mean age of 60.1 ± 7.6 years, resulting in a prevalence of approximately 9.7%. Only two of these seven relatives showed clinical signs of intermediate AMD, while most showed small preclinical changes such as druplets. All but one of these seven were former or current smokers with a mean body mass index (BMI) of 32.8 ± 9.4 as compared to relatives without SDD who had a mean age of 51.4 ± 14.5 years, mean BMI of 26.8 ± 7.0, and never smoked in 46%.

In participants with data on SDD area, the mean percentage of area affected by SDD was 68% and 66% for the right and left eye, respectively, with large differences between index patients and relatives. Further details and population characteristics can be found in Table 1.

**Table 1:** Population characteristics

Variable	*N*	Total (*N* = 195^1^)	Index patients (*N* = 123^1^)	Relatives (*N* = 72^1^)	*P*-value^2^
Age	195	70.64 (17.50)	81.41 (7.23)	52.23 (14.22)	< 0.001
Sex	195				0.10
Female		123 (63%)	83 (67%)	40 (56%)	
Male		72 (37%)	40 (33%)	32 (44%)	
BMI	185	26.18 (5.72)	25.45 (4.22)	27.34 (7.38)	0.15
Smoking	185				0.005
Never		96 (52%)	65 (58%)	31 (43%)	
Former		58 (31%)	37 (33%)	21 (29%)	
Current		31 (17%)	11 (9.7%)	20 (28%)	
Arterial hypertension	186	114 (61%)	84 (74%)	30 (42%)	< 0.001
Diabetes	186	30 (16%)	19 (17%)	11 (15%)	0.8
SDD either eye	195				< 0.001
Yes		130 (67%)	123 (100%)	7 (9.7%)	
Percentage area SDD OD	107	68.01 (22.72)	69.79 (20.65)	31.60 (34.30)	0.013
Percentage area SDD OS	115	66.40 (22.90)	68.33 (20.66)	12.80 (17.64)	0.001
BCVA OD^3^	178	0.8 (0.85)	0.4 (0.53)	1.25 (0)	< 0.001
BCVA OS^3^	175	0.8 (0.85)	0.5 (0.55)	1.25 (0.25)	< 0.001
LLVA OD	138	0.48 (0.32)	0.23 (0.16)	0.71 (0.25)	< 0.001
LLVA OS	137	0.46 (0.29)	0.24 (0.16)	0.68 (0.23)	< 0.001

*BMI* body mass index, *SDDs* subretinal drusenoid deposits, *OD* oculus dexter, *OS* oculus sinister, *BCVA* best-corrected visual acuity, *LLVA* low-luminance visual acuity

^1^Mean (SD); *N* (%)

^2^Wilcoxon rank-sum test; Pearson’s chi-squared test

^3^Median (IQR)

In logistic regression of the total cohort, we found an increased odds ratio (OR) for presence of SDD with older age (per year OR 1.27, 95% confidence interval (CI) 1.17 to 1.41, *P* < 0.001). In a subgroup analysis of relatives, we found a trend of higher OR of SDD with higher BMI (1.16, 95%CI 1.00 to 1.45, *P* = 0.08). In linear regression, we found older age to be associated with a larger percentage of area affected by SDD (per year beta = 0.92, 95% CI 0.13 to 1.72, *P* = 0.02). We found no association with the other assessed potential determinants.

### Genetic analyses

**Fig. 1:**
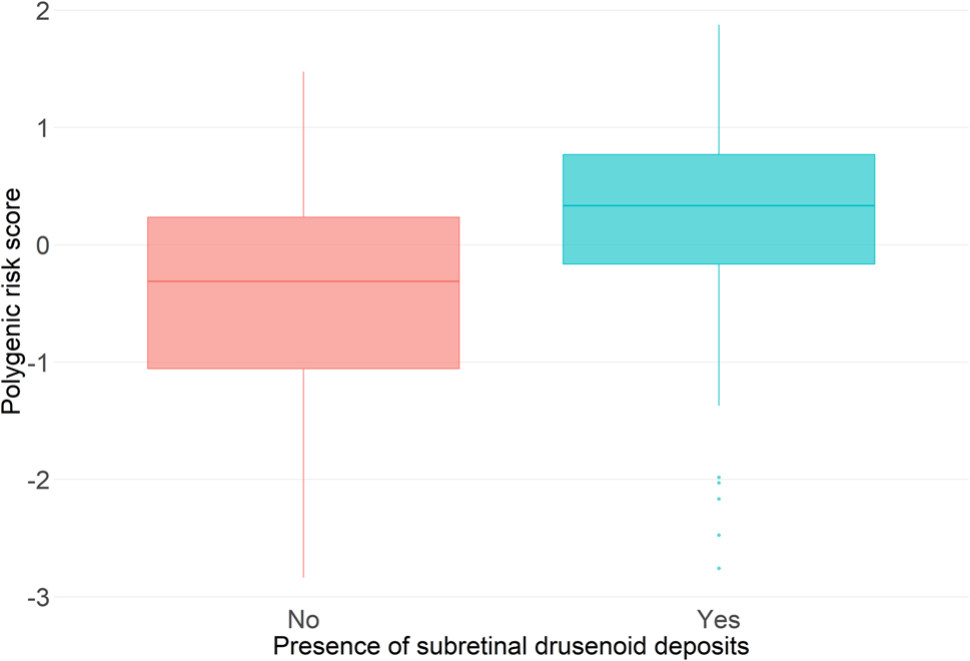
Boxplot of polygenic risk score (PRS) in individuals with and without subretinal drusenoid deposits (SDDs)

For AMD, we found a higher PRS to be borderline associated with a more likely AMD presence (per unit OR 4.99, 95%CI 0.95 to 26.23, *P* = 0.057) in multivariable logistic regression models. For SDD, we observed a trend of higher PRS in individuals with SDD (Fig. 1). This trend, however, was not found in the multivariable logistics regression model (OR 1.87, 95% CI 0.61 to 5.76, *P* = 0.27), in which only age was associated with presence of SDD. Similarly, we found only age and not the PRS to be associated with the area of SDD in the multivariable linear regression models (data not shown). We furthermore found no individual SNP to be associated with presence of SDD. Supplemental figure 2 presents a Manhattan plot for the 53 tested SNPs.

## Discussion

Our study reports a prevalence estimate for SDD in first-degree relatives—mostly children—of patients with SDD and identifies age as main determinant of SDD presence. While included relatives were on average younger than index patients, those relatives presenting with SDD were older than those without. Moreover, we found an association of older age with larger retinal en face area affected by SDD and a trend of higher AMD polygenic risk score in individuals with SDD. Our study indicates a heritable trait of SDD which, however, ought to be re-evaluated in longitudinal studies with larger sample size.

First reports of SDD date back to the early 1990s [26], and we know by now that they are distinct from regular drusen and located anterior to the retinal pigment epithelium [3, 13].

Particularly, their uniform dot and ribbon-like appearance across affected patients in the different imaging modalities have given rise to various pathogenetic hypotheses, ranging from widespread inflammation [27] to dependencies on choroidal vasculature [28]. Yet, the underlying pathomechanisms still remain to be elucidated.

Our detected prevalence of 9.7% in relatives is lower than in previous studies using SD-OCT images in the general population, which is mainly explained by the much lower mean age in our cohort. The Montrachet study reported a prevalence of 18% based on SD-OCT images in a cohort with an average age of 82 years [13], and data from the Alienor study suggest a prevalence of 13% in individuals aged 77 years or older [11]. Given the age difference over 25 years, we interpret our detected prevalence to be relatively high nonetheless.

We found age to be the main driver of all detected associations in our cohort. Aging is a proxy for a range of systemic processes that can cause metabolic deterioration over the life span and potentially cause multifactorial diseases such as AMD or cardiovascular disease (CVD) [29]. To date, we do not fully understand the exact underlying pathomechanisms causing these deteriorations. As mentioned, various pathways such as the lipid metabolism or inflammation seem to play a role in AMD and potentially SDD development. The complexity of various simultaneous metabolic changes, however, makes it particularly difficult to identify specific molecular causes.

Our study confirms the previously reported association of SDD with age [13, 15], and we found that only two relatives with SDD had AMD-defining retinal lesions such as intermediate/large drusen or pigmentary abnormalities but showed small alterations such as druplets. This may indicate an independency of SDD from AMD (particularly in the early course of disease) and/or suggest that SDD could be a precursor in those at risk. Given the age difference between index patients and relatives, relatives may have been too young to present large drusen and may still develop these in the course of their lives.

We found a trend of higher BMI in relatives with SDD, which is in agreement with previous studies and may mirror the metabolic deterioration in SDD formation [4, 11, 16, 17]. The fact that we only found the effect in the younger relatives may indicate that BMI has a larger impact at a younger age when fewer other risk factors are present.

As expected, AMD patients showed a higher AMD PRS, which was only borderline statistically significant due to lack of statistical power. Moreover, individuals with SDD seemed to have a higher PRS, which, however, did not remain associated in the multivariable models likely due to lack of statistical power. We hypothesize that the entanglement between AMD and SDD may have created noise in the data and hence lowered statistical power of the AMD PRS to discriminate those with and without SDD. This is in line with previous literature that reported partly contradicting results about the association of SDD with AMD risk genes [3]. While some studies report an association with polymorphisms in the gene for *CFH* [30, 31] and the *ARMS2* gene [32, 33], other studies could not find any association [34–36].

Our results support that genetic risk contributes to SDD formation. The incidence and onset of AMD in those with SDD, however, remain to be elucidated in larger longitudinal studies. Based on our data, clinical implications include recommendations to screen relatives of persons affected by both SDD and AMD. Moreover, it remains essential to inform patients and their relatives of prevention measures such as smoking cessation, regular physical activity, and an adequate diet [29, 37].

The direct clinical consequences of SDD presence for the individual still remain to be elucidated. While functional impairments such as scotopic deterioration may occur over time, it remains unclear whether these are directly caused by SDDs or whether presence of SDD indicates a more general metabolic worsening that also cause scotopic impairment.

The strengths of this study include a standardized assessment protocol for patients and relatives including interviews, a full ophthalmic examination and high-resolution retinal imaging. Examinations were conducted by trained staff, and grading for SDD was performed according to a previously described classification [3] by two graders (BJH and MMM) with senior arbitration (RPF) in case of disagreement. SDDs were semi-quantitatively graded by area of SDD in addition to the commonly used binary grading. The genetic analyses are based on state-of-the-art sequencing techniques, and we investigated all previously described loci [24]. To our knowledge, this is the first study to primarily investigate presence of SDD and genetic predisposition in close relatives of patients with SDD.

However, several limitations need to be acknowledged. Our study may underlie a selection bias. Index patients were selected based on presence of SDD at their regularly scheduled visit at the outpatient clinic, mainly for neovascular AMD. Hence, our index patients are potentially more likely to have more severe AMD phenotypes than individuals with SDD in the general population. Moreover, included relatives may be more prone to participate due to, e.g., severe ophthalmic symptoms in their related index patient. However, we think that any bias likely would lead to stronger associations as the inclusion of more severe cases may indicate a higher genetic risk of AMD and potentially SDDs and hence increased chances to detect associations. We have no information on reasons for index patients not to contact their relatives. Main reasons for contacted relatives not to participate were long-distance drives to the clinic, no intact relationships to their relative index patient, and generally no interest in participating in research.

The included relatives were on average younger than our index patients, which likely is one of the main drivers for the relatively low prevalence. At the same time, early structural changes towards SDD development in relatives might not yet be detectable with current available image resolution in IR and SD-OCT imaging leading to an underestimated prevalence of SDDs in our study cohort. In addition, inclusion of older relatives may more likely pick up those who will develop SDD and/or AMD within the later course of their life. We tried to overcome this shortcoming by including the AMD PRS which is independent of age and found that it was on average higher in index patients as compared to relatives indicating that not all relatives have similar genetic predisposition (supplemental figure 3). Yet, we lack genetic data from a control group of individuals without SDD and their respective relatives. Moreover, differences in dilation and presence of cataract between index patients and relatives may have introduced noise in our data. Lastly, the moderate sample size did not provide large statistical power for genetic analyses such as the SNP-wise modeling within the Manhattan plot leaving us presumably underpowered for genetic discoveries. Hence, we likely missed associations in the multivariable models, underscoring the need for further large-scale analyses.

In conclusion, our study indicates a hereditary predisposition for SDDs in relatives of patients with SDD and confirms the strong association with age as important non-modifiable risk factor. Based on our results, relatives of patients with SDD ought to be closely monitored for retinal alterations, particularly at an older age. Our study lays foundation for further longitudinal studies with larger sample size to evaluate the genetic predisposition of SDD and confirm and extend or refute our findings.

### Supplementary information

ESM 1 (JPG 163 kb)

ESM 2 (PNG 165 kb)

ESM 3 (PNG 56 kb)
