# Supplementary figures and images for: Prevalence and determinants of subretinal drusenoid deposits in patients’ first-degree relatives
Source: Graefes Arch Clin Exp Ophthalmol. 2023 Sep 6;262(1):53–60. doi: 10.1007/s00417-023-06221-y (PMC10805990; doi:10.1007/s00417-023-06221-y)

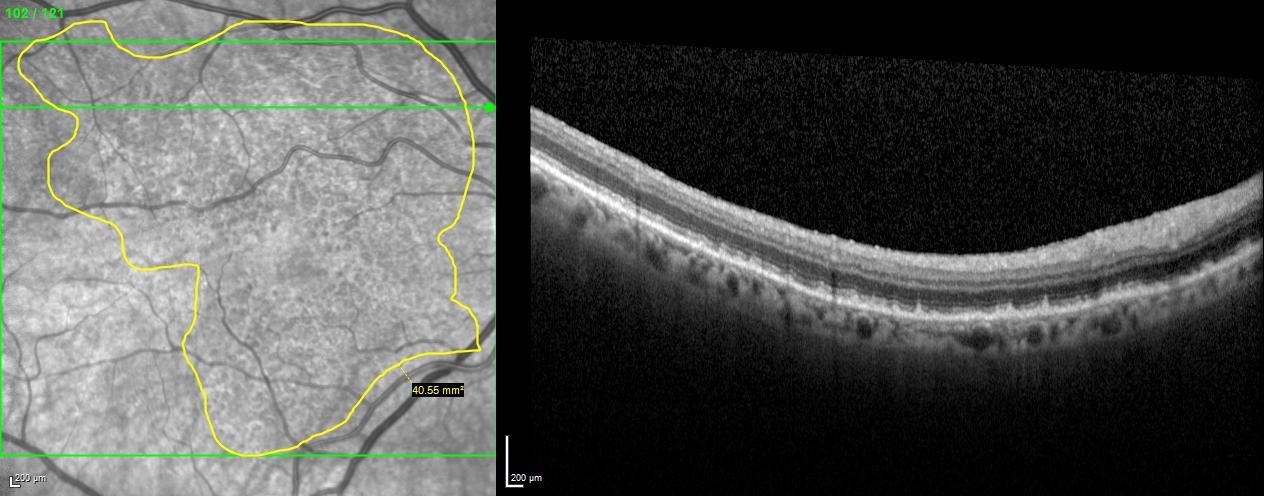

Supplement: Supplementary file 1 — (JPG 163 kb) [file 417_2023_6221_MOESM1_ESM.jpg]

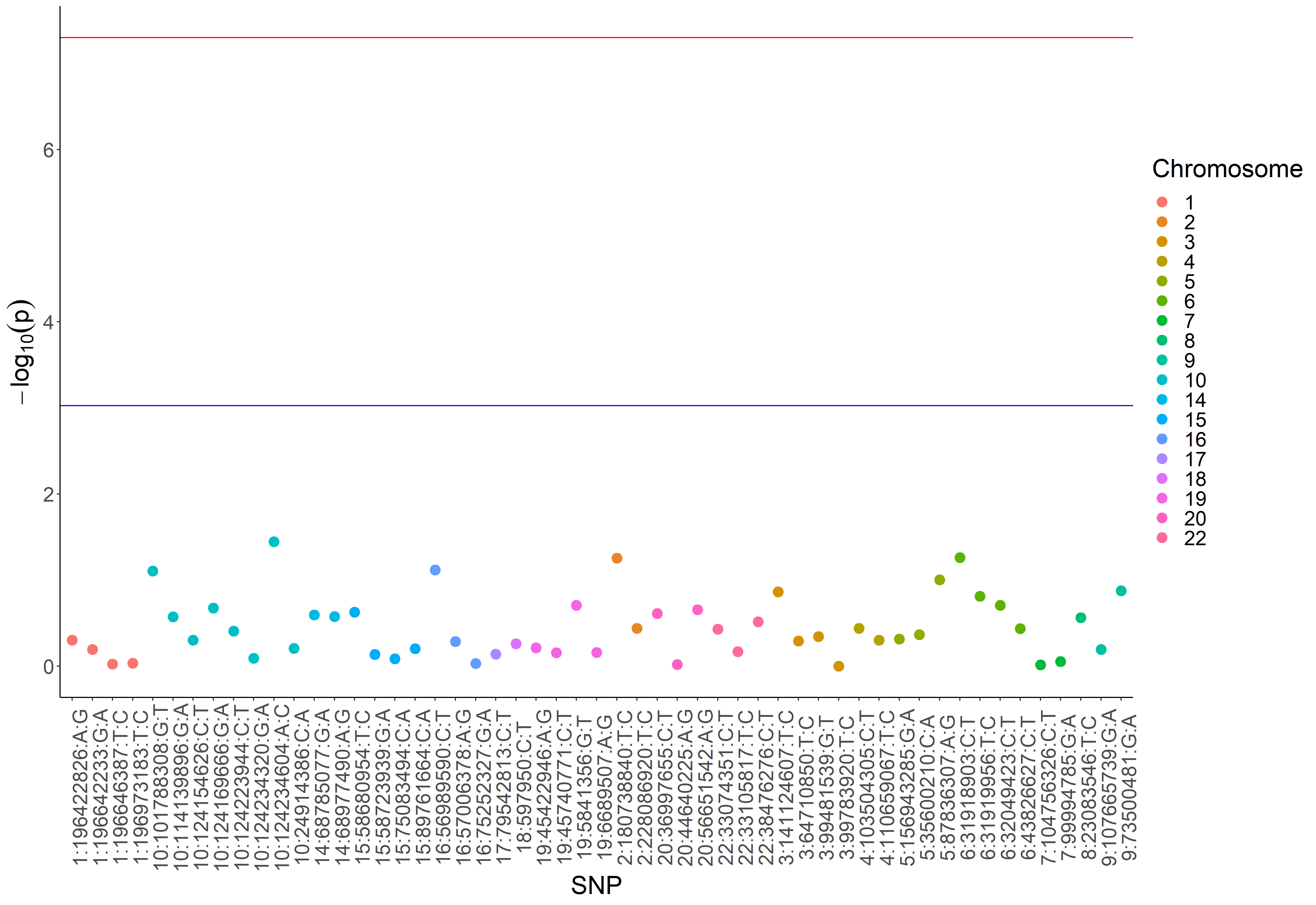

Supplement: Supplementary file 2 — (PNG 165 kb) [file 417_2023_6221_MOESM2_ESM.png]

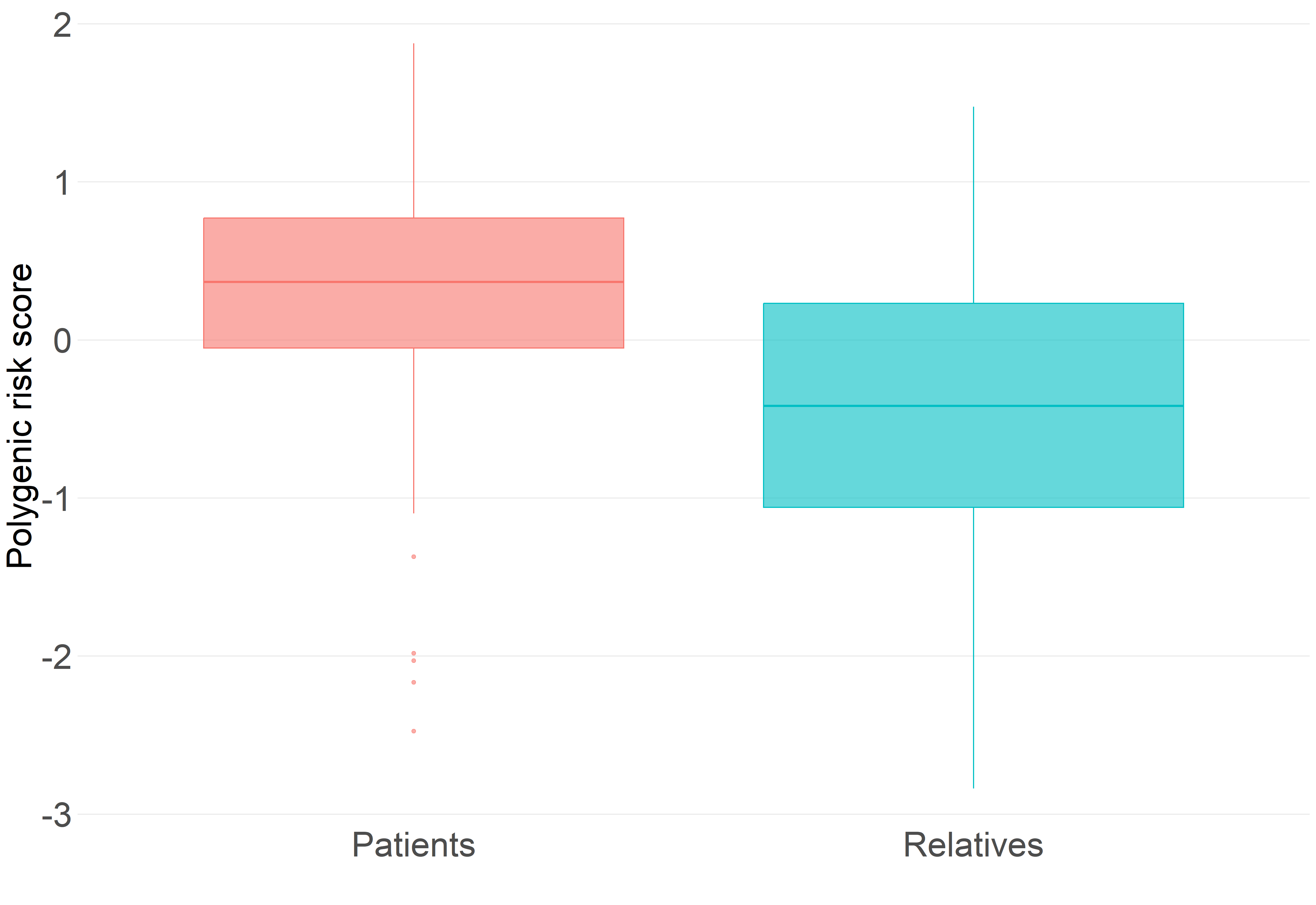

Supplement: Supplementary file 3 — (PNG 56 kb) [file 417_2023_6221_MOESM3_ESM.png]
